# Cumulative Live Birth Rates After the First ART Cycle Using Flexible GnRH Antagonist Protocol vs. Standard Long GnRH Agonist Protocol: A Retrospective Cohort Study in Women of Different Ages and Various Ovarian Reserve

**DOI:** 10.3389/fendo.2020.00287

**Published:** 2020-05-08

**Authors:** Wanlin Zhang, Duo Xie, Hengde Zhang, Jianlei Huang, Xifeng Xiao, Binrong Wang, Yafei Tong, Ye Miao, Xiaohong Wang

**Affiliations:** ^1^Department of Obstetrics and Gynecology, Reproductive Medical Center, The Second Affiliated Hospital, Air Force Military Medical University, Xi'an, China; ^2^Department of Obstetrics and Gynecology, 986 Hospital of Air Force, Xi'an, China; ^3^Department of Anesthesiology, Xi'an International Medical Center Hospital, Xi'an, China

**Keywords:** *in vitro* fertilization, ovarian reserve, GnRH antagonist, GnRH agonist, cumulative live birth rate

## Abstract

**Objective:** To compare the cumulative live birth rates (cLBRs) after the first assisted reproductive technology (ART) cycle using flexible gonadotropin releasing hormone (GnRH)-antagonist protocol vs. standard long GnRH agonist protocol for controlled ovarian stimulation (COS) in infertile women with different ages and ovarian reserve.

**Methods:** Women who underwent ART treatment at our center between June 1st, 2015 and December 31st, 2018 were screened. Among them, only women who underwent their first COS cycle with flexible GnRH antagonist protocol or standard long GnRH agonist protocol were included in this study. The main outcome measurement was cLBR.

**Results:** A total of 4,402 patients were eligible for the analysis, of whom, 2,762 patients used the GnRH agonist protocol and 1,640 patients used the GnRH antagonist protocol. The cLBRs of women in the antagonist protocol group and long agonist protocol group were 45.3 and 50.0%, respectively. Subgroup multivariable regression analysis showed that, in patients with low ovarian reserve (AFC ≤ 7), the cLBR was significantly lower in the antagonist group than in the long agonist protocol group [OR (95% CI) 0.62 (0.41, 0.94)], which effect was more robust in younger patients (<30 y) [OR (95% CI) 0.29 (0.11, 0.74)]. The analysis also revealed remarkably lower cLBR in patients above 40 years regardless of their AFC, although the difference was not statistically significant. However, in patients with high ovarian reserve (AFC >24), the cLBR was higher in cycles with antagonist protocol than with the long agonist protocol [OR (95% CI) 1.43 (0.96, 2.12)], and the effect was of statistical significance in younger patients (< 30 y) [OR (95% CI) 1.78 (1.07, 2.96)].

**Conclusion:** The present study suggests that the flexible GnRH antagonist protocol might not be suitable for patients with low ovarian reserve (AFC ≤ 7) or patients aged over 40 years. However, flexible GnRH antagonist protocol might be strongly recommended for patients under 30 years old and with high ovarian reserve (AFC > 24). For the rest groups of patients in the present cohort, antagonist protocol was slightly favored because it had lower OHSS in general and in patients with poly-cystic ovarian syndrome (PCOS) according to previous publications.

## Introduction

*In vitro* fertilization and embryo transfer (IVF-ET) as the most effective treatment for infertility has been widely used worldwide. IVF is a multi-step process starting from ovarian stimulation with gonadotropins, then oocyte retrieval with subsequent fertilization procedures in the laboratory, and embryo culture followed by embryo transfer into the uterus. The controlled ovarian stimulation (COS) is the first step with the purpose of inducing maturation of multiple oocytes, and hence maximizing the chance of achieving successful pregnancy. However, multi-follicular development often results in premature elevation of estradiol followed by early luteinizing hormone (LH) surge and premature luteinization, which has been shown to affect 12.3–46.7% of fresh IVF cycles, and there is accumulating evidence confirming its negative effect on the overall success rates (1, 2). Two artificial gonadotropin releasing hormone (GnRH) analogs, GnRH agonist and GnRH antagonist, have been applied to address these issues, both of which are effective in blocking premature LH surge (3–5).

GnRH agonist was the first GnRH analog introduced into COS to prevent the premature elevation of endogenous LH in 1984 (6). It competitively binds with the GnRH receptors in pituitary to slowly desensitize the pituitary. Short-acting GnRH agonist long protocol, also known as “long protocol” which starts from mid-luteal phase, has been the gold standard for pituitary downregulation method in COS worldwide nowadays, especially in young normo-gonadotropic women. The long protocol has plenty of advantages, such as maintaining stable and low LH and progesterone (P) levels throughout the stimulation phase, synchronized follicular development, good number of retrieved oocytes and short learning curve (6–8). But this suppression is related to clear disadvantages including the initial flare-up and menopausal symptoms (9). GnRH antagonist was developed about 40 years ago, but wasn't widely applied in clinical practice until recently (3). It instantly blocks the pituitary LH secretion without any flare-up effect and is proved to be with shorter treatment duration, less use of gonadotropic hormones, improved patient acceptance, but with fewer follicles and oocytes when compared with standard long protocol in various clinical studies (4, 5, 10, 11). Advantages of GnRH antagonist as mentioned above, numerous clinical trials and meta-analyses (12, 13) based on the studies comparing GnRH agonist protocol and antagonist protocol have showed a consistent conclusion that GnRH antagonist protocol results in similar live birth rate (LBR) but with significantly lower incidence of any grade OHSS in IVF regardless of treated population (14). Even in population with polycystic ovary syndrome (PCOS), clinical pregnancy rate was also comparable between the group receiving GnRH antagonist protocol and the other receiving long protocol, but the OHSS rate was significantly lower in the GnRH antagonist protocol group (15, 16).

With these results, the debate regarding the clinical pregnancy outcomes of the two protocols seems to be closing. Does it mean it is time to change the standard COS protocol from GnRH agonist long protocol to GnRH antagonist protocol? In clinical experience, there's no “one size fits all.” No single COS protocol is suitable for all populations. Regretfully, consistent conclusions across studies comparing the two protocols in different populations failed to be drawn. Lambalk et al. (17) conducted a meta-analysis including 50 randomized controlled trials (RCTs) to compare the effectiveness and safety between the two protocols in different patient types. They concluded that although GnRH antagonist protocol led to less OHSS occurrence but also lower ongoing pregnancy rate (OPR) when compared with standard long agonist protocol in general population, the fact that 1 in 40 reduction of OHSS occurrence costed 1 in 28 reduction of ongoing pregnancy was not convincing enough for us to make the decision of changing the GnRH analogs from agonist to antagonist in the general population. Nonetheless, the authors suggested the standard use of GnRH antagonist in PCOS patients and poor responders due to less OHSS but comparable OPR in GnRH antagonist protocol group in these two populations. Grow et al. conducted a large retrospective study (*n* = 203,302 fresh, autologous cycles) to compare the clinical outcomes between GnRH antagonist and long agonist protocols in good-prognosis patients and sub-group patients who received selective single embryo transfer. After adjusting several confounding factors, they found that GnRH antagonist protocol resulted in lower LBR in the general study population as well as in the sub-group population (18). Although these studies tried to optimize the use of these two protocols in a population-specific way, the stratification was not sufficient enough, and the outcomes were not consistent. Given this fact, the effort to investigate the patient-tailored COS strategy is still warranted.

The objective of the present study was to compare the cLBR between patients receiving flexible GnRH antagonist protocol and standard GnRH agonist long protocol during IVF in infertile women of different ages with various ovarian reserve, and to make one step further to provide some evidence for the more appropriate clinical application.

## Materials and Methods

### Patient Selection

Patients who underwent ART treatment for infertility in our clinic from June 1st, 2015 to December 31st, 2018 were unselectively and consecutively screened according to the inclusion and exclusion criteria. Only the first COS cycles treated with flexible GnRH antagonist protocol and short-acting GnRH agonist long protocol starting from luteal phase of the menstrual cycle for pituitary down regulation were selected. Other COS protocols or second or further cycles were excluded from the cohort. To minimize the selection bias, women with the diagnosis of recurrent spontaneous abortion, defined as three or more consecutive miscarriages occurring before 20 weeks post-menstruation (19), were also excluded from the cohort.

### Clinical Characteristics

Baseline demographic parameters including female age (years), body mass index (BMI) (kg/m^2^), duration of infertility, infertility factors and infertility diagnosis were collected Baseline IVF-specific characteristic data including basal plasma follicle-stimulating hormone (FSH) level (mIU/mL), basal estradiol level (pg/mL), basal LH level (mIU/mL) and basal antral follicle count (AFC), all measured on menstrual cycle Day 2 or Day 3, were collected. Anti-Mullerian hormone (AMH) measured on any day of a menstrual cycle was also documented. COS parameters documented for all patients included duration of ovarian stimulation (days), total dose of gonadotropins (IU), peak estradiol level (pg/mL), progesterone level (ng/mL), LH level (mIU/mL) on human chorionic gonadotrophin (hCG) trigger day, number of oocytes retrieved and number of mature oocytes. The documented embryo variables included fertilization method, number of normal fertilized oocytes, number of usable embryos, number of good quality embryos and number of patients who underwent preimplantation genetic testing (PGT). The main outcome measurement was cLBR, which was defined as the number of patients with at least one live birth divided by the number of all eligible patients underwent the first oocyte retrieval cycle within the study period. Data on embryo transfer cancelation for preventing OHSS was also briefly described.

### COS Protocols

COS protocols were performed as reported in one of our previous articles (20). Briefly, for the short-acting GnRH agonist long protocol, GnRH agonist[Triptorelin Acetate (Diphereline®)]0.1 mg by subcutaneous injection was administered daily starting from the mid-luteal phase of the menstrual cycle, lasting for 10–14 days until the pituitary down-regulation was confirmed, then the ovarian stimulation with gonadotropin (Gn) commenced. For the flexible GnRH antagonist protocol, ovarian stimulation began on the second day of the menstrual cycle and the antagonist(Cetrorelix Acetate, Cetrotide®, 0.125–0.25 mg daily) administration started as soon as the leading follicles reached the size of 14 mm or larger in diameter, and the antagonist administration continued until the day of hCG administration for trigger.

### Statistical Analysis

Categorical variables are expressed as number (n) and percentage (%) and continuous variables are expressed as mean ± standard deviation (SD). Normality was checked through Shapiro-Wilk normality test in addition to visual inspection of the distribution. Characteristics of the study population in the GnRH agonist long protocol group and the flexible GnRH antagonist protocol group were described separately. Student's *t*-test was applied for the primary comparison between the two groups. Multivariable logistic regression analysis was performed to compare the cLBRs between the two groups. Female age, infertility factors, infertility duration, infertility diagnosis, female BMI and fertilization method were adjusted in the final adjusted model. Additional analyses was performed stratified by AFC (AFC ≤ 7 vs. >7; ≤ 24 vs. >24), female age (female age <30 years vs. ≥30 years, <40 y vs. ≥ 40 years) and the combination of the above two parameters. Confounding factors as mentioned above were all adjusted in the stratification analyses except the stratification factor itself. Interactions between stratification factors and the COS protocols were also tested. Because some data were missing for confounding factors of BMI and infertility duration (missing data in 72 cases and 10 cases, respectively), multivariable regression analyses for cohort with missing data, cohort with complete data and cohort with multiple imputation for the missing data were also performed, respectively, to understand the robustness of the outcome. Unadjusted and adjusted odds ratios (ORs) with 95% confidence intervals (CIs) were calculated based on the corresponding models. Statistical significance was reached at *P* < 0.05 and all statistical analyses were performed using the statistical software packages R (http://www.R-project.org, The R Foundation) and EmpowrStats software (http://www.empowerstats.com, X&Y Solution, Inc., Boston, MA).

## Results

### Characteristics of the Studied Population

**Figure 1:**
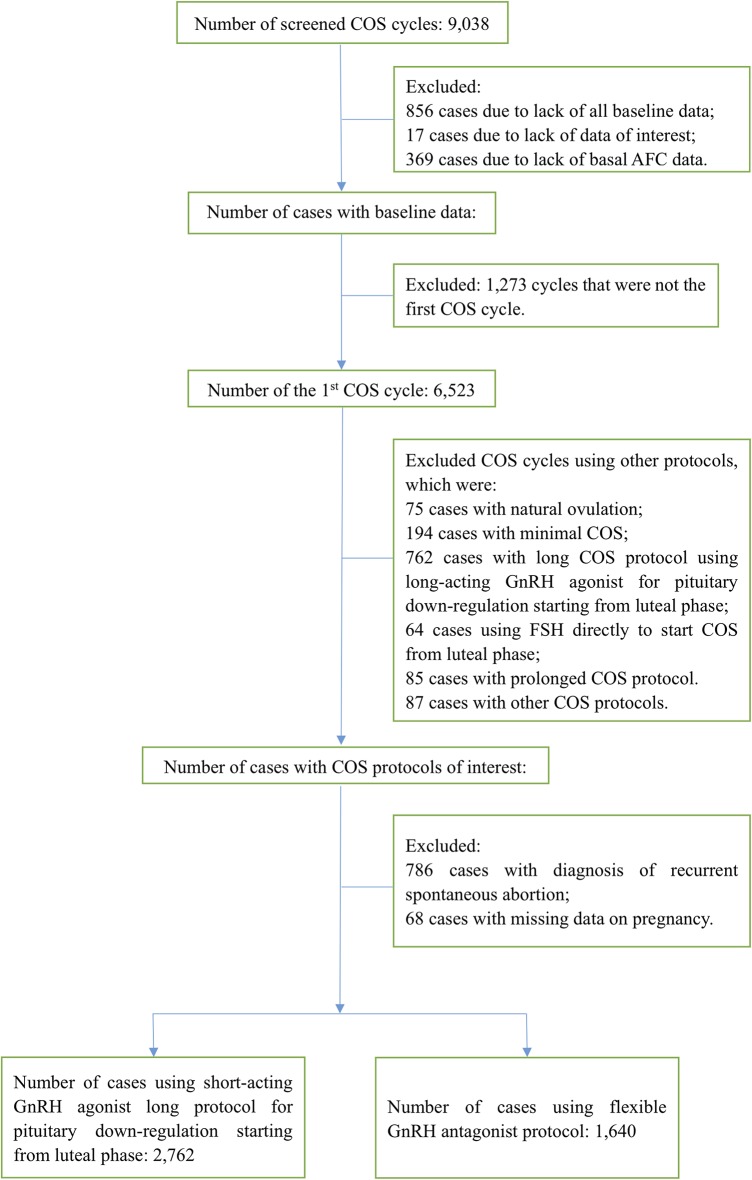
Flow chart of patient disposition.

A total of 9,038 COS cycles during the study period were screened for inclusion and exclusion. Among these, 4,636 (51.3%) cycles were excluded due to any of the following reasons: inadequate data, not the first stimulation cycle and COS with other protocols. Patients with the diagnosis of repeated spontaneous abortion were also excluded from the cohort. Finally, the remaining 4,402 patients were eligible for analysis. Details of patient selection is shown in the patient disposition flow chart (Figure 1). The cLBR was 72.9% (2,138/2,931) for patients who underwent the embryo transfer cycle(s), and 48.6% (2,138/4,402) for the overall study population. There were 1,296 (29.4%) patients underwent the fresh embryo transfer cycle and 2,286 patients underwent the first frozen embryo transfer cycle. Of patients underwent the first frozen embryo transfer cycle, 1,632 patients were with freeze all strategy in the previous stimulation cycle and 654 patients failed to be pregnant in the fresh embryo transfer cycle. There were 29.3% (802/2,734) patients and 30.1% (494/1,640) patients underwent the fresh embryo transfer cycle in GnRH agonist and antagonist group, respectively, and the difference was not statistically significant. The number of patients underwent the second frozen embryo transfer cycle was 346, including patients who failed to get pregnant in the previous embryo transfer cycle(s). Only 29 patients underwent the third frozen embryo transfer cycle. The remaining 1,471 patients hadn't had their embryos transferred at all during the study period. The following comparison of the cLBRs between the GnRH agonist long protocol group and the GnRH antagonist protocol group was based on the data of the overall study population.

In the cohort, 2,762 patients received the GnRH agonist long protocol and 1,640 patients received the GnRH antagonist protocol. A total of 126 (2.9%) patients underwent PGT, including 109 (3.9%) patients in the GnRH agonist protocol group and 17 (1.0%) in the flexible GnRH antagonist protocol group. Demographics of the overall population and population by group, baseline infertility characteristics, COS parameters, embryo parameters and cLBR of the studied cohort were compared and shown in Table 1.

**Table 1:** Demographic characteristics of the study population.

		**GnRH agonist**	**GnRH antagonist**	***P***
*n*	4,402	2,762	1,640	
Female age (y)	30.7 ± 4.5	30.2 ± 4.1	31.6 ± 5.0	<0.001
BMI (kg/m^2^) (data missing, *n* = 72)	22.4 ± 3.2	22.3 ± 3.3	22.5 ± 3.2	0.012
Infertility diagnosis (*n* [%])				0.329
Primary infertility	2,353 (53.5)	1,492 (54.0)	861 (52.5)	
Secondary infertility	2,049 (46.5)	1,270 (46.0)	779 (47.5)	
Infertility duration (y) (*n*[%]) (data missing, *n* = 10)	4.1 ± 3.2	3.9 ± 3.1	4.3 ± 3.5	0.002
Infertility factors (*n*[%])				<0.001
Pelvic and tubal factors	2,760 (62.7)	1,816 (65.7)	944 (57.6)	
Ovulation disorder	113 (2.6)	64 (2.3)	49 (3.0)	
Endometriosis	69 (1.6)	42 (1.5)	27 (1.6)	
Male factor	637 (14.5)	426 (15.4)	211 (12.9)	
Female and male factors	255 (5.8)	170 (6.2)	85 (5.2)	
Unexplained	568 (12.8)	244 (8.9)	324 (19.7)	
Basal FSH (mIU/mL)	7.7 ± 2.9	7.4 ± 2.6	8.1 ± 3.3	<0.001
Basal LH (mIU/mL)	5.1 ± 4.9	5.3 ± 5.8	4.6 ± 3.0	<0.001
Basal estradiol (pg/mL)	63.2 ± 53.8	66.1 ± 56.0	58.4 ± 49.5	<0.001
AMH (ng/mL)	3.4 ± 2.8	3.7 ± 2.8	3.0 ± 2.8	<0.001
Number of basal AFC	15.1 ± 7.4	15.9 ± 7.1	13.7 ± 7.8	<0.001
Duration of Gn (d)	11.6 ± 2.4	11.8 ± 2.5	11.2 ± 2.3	<0.001
Total dose of Gn (IU)	2,370 ± 1230	2,287.5 ± 1230	2,512.5 ± 1207.5	<0.001
Estradiol level on hCG trigger day (pg/mL)	3,599.5 ± 1709.2	3,972.9 ± 1595.8	2,944.6 ± 1704.9	<0.001
LH level on hCG trigger day (mIU/mL)	10.7 ± 188.3	11.3 ± 208.1	9.6 ± 147.5	0.79
Progesterone level on hCG trigger day (ng/mL)	1.0 ± 0.5	0.9 ± 0.5	1.0 ± 0.5	<0.001
Number of retrieved oocytes	12.3 ± 6.1	13.1 ± 5.8	11.0 ± 6.3	<0.001
Number of MII oocytes	10.1 ± 5.4	10.8 ± 5.2	9.1 ± 5.6	<0.001
Fertilization method				<0.001
IVF (*n*[%])	2996 (68.1)	1858 (67.3)	1130 (69.4)	
ICSI (*n*[%])	1085 (24.6)	725 (26.2)	360 (22.0)	
Half IVF + half ICSI (*n*[%])	55 (1.2)	27 (1.0)	28 (2.7)	
IVF + rescue ICSI (*n*[%])	266 (6.0)	152 (5.5)	114 (7.0)	
Number of 2PN embryos	8.0 ± 4.6	8.5 ± 4.5	7.1 ± 4.7	<0.001
Number of usable embryos	4.7 ± 3.2	5.1 ± 3.2	4.1 ± 3.0	<0.001
Number of good quality embryos	4.0 ± 3.1	4.3 ± 3.1	3.4 ± 2.9	<0.001
Number of PGT cases	126 (2.9)	109 (3.9)	17 (1.0)	<0.001
Number of embryo transfer cycle cancelation due for OHSS prevention (data missing, *n* = 1,263) (*n*[%])	1,422 (45.2)	1,005 (49.6)	417 (37.3)	<0.001
cLBR (n[%])	2,138 (48.6)	1,395 (50.0)	743 (45.3)	<0.001

*AFC, antral follicular count; AMH, Anti mullerian hormone; BMI, body mass index; FSH, follicle stimulating hormone; Gn, gonadotropinh; hCG, human chorionic gonadotropin; ICSI, intracytoplasmic single sperm injection; IVF, in vitro fertilizationl; LH, luteinizing hormone; OHSS, ovarian hyperstimulation syndrome; PGT, preimplantation genetic testing. A summary of the demographic characteristics of the study population and a crude comparison of the clinical outcomes between the two GnRH analogs. Student's t test was applied for the primary comparison between the two groups. Statistical significance was reached at P < 0.05*.

As shown in Table 1, compared to the GnRH agonist long protocol group, the GnRH antagonist protocol group had shorter duration of ovarian stimulation, lower peak estradiol level on the hCG trigger day and smaller number of fresh embryo transfer cycle cancellations due to OHSS risk (37.3 vs. 49.6%, [missing data, *n* = 1,263]), all with statistical significance. Nonetheless, the number of retrieved oocytes, the number of mature oocytes, the number of normal fertilized oocytes, the number of usable embryo and the number of high-quality embryos were significantly smaller in the GnRH antagonist group than in the GnRH agonist group. The cLBR was also significantly lower in the GnRH antagonist group than in the GnRH agonist group (45.3 vs. 50.0%).

### Outcomes of Stratification Analysis With Multivariable Logistic Regression

With all confounding factors taken into account [female age, female BMI, infertility duration, infertility diagnosis, infertility factors and method of fertilization], the results of the multivariable regression analysis failed to show any difference in the cLBR between the two groups [OR (95% CI), 1.00 (0.87, 1.14)] (Table 2).

**Table 2:** Comparison of cLBRs of flexible GnRH antagonist protocol vs. GnRH agonist long protocol using multivariable regression analysis in subgroup patients with different AFCs and of different ages.

	**Non-adjusted**	***P***	**Adjusted**	***P***	***P* for interaction**
Basal AFC ≤ 7	*0.50 (0.35, 0.73)*	*0.0003*	*0.62 (0.41, 0.94)*	*0.026*	*0.013*
Basal AFC >7, ≤ 24	0.99 (0.86, 1.15)	0.902	1.02 (0.88, 1.19)	0.805	
Basal AFC >24	1.35 (0.94, 1.96)	0.109	1.43 (0.96, 2.12)	0.079	
Female age <30 y	0.98 (0.81, 1.19)	0.828	1.01 (0.82, 1.23)	0.952	0.526
Female age ≥30 y, <40 y	0.89 (0.75, 1.06)	0.191	0.92 (0.77, 1.10)	0.347	
Female age ≥40 y	0.67 (0.26, 1.73)	0.412	0.58 (0.21, 1.58)	0.288	
Total	0.95 (0.84, 1.08)	0.424	1.00 (0.87, 1.14)	0.985	

*The cLBR was not significantly different between the two groups after adjusting for confounders of female age, female BMI, infertility duration, infertility diagnosis, infertility factors and method of fertilization in general population [adjusted OR 1.00 95%CI (0.87, 1.14)]. However, whether adjusting for confounders or not, a significant decrease of cLBR was seen in GnRH antagonist group for patients with basal AFC <7 [non-adjusted OR 0.50 95%CI (0.35, 0.73), adjusted OR 0.62 95%CI (0.41, 0.94)].P for interaction test between GnRH analogs and AFCs was statistical significant, indicating that patients with basal AFC <7 might be really a special population that should not be treated with GnRH antagonist. Significant changes of cLBR in other subgroup patients were not seen. The italic values represent that the differences are statistically significant*.

However, when the study population was stratified by AFC (AFC ≤ 7 vs. AFC >7; ≤ 24 vs. AFC > 24), which is a primary marker of ovarian reserve besides female age, the results of multivariable regression analysis showed that in patients with low ovarian reserve (AFC ≤ 7), the flexible GnRH antagonist protocol resulted in significantly lower cLBR than the GnRH agonist long protocol [OR (95% CI), 0.62 (0.41, 0.94)]. For patients with AFC between 7 and 24, the flexible GnRH antagonist protocol resulted in similar cLBR with the GnRH agonist long protocol [OR (95% CI), 1.02 (0.88, 1.19)]. Interestingly, in patients with AFC > 24, the flexible GnRH antagonist protocol resulted in favorable cLBR compared with GnRH agonist long protocol [OR (95% CI), 1.43 (0.96, 2.12)], although the difference didn't reach the statistical significance, the effect value was considerable. The interaction test results revealed that AFC might be a remarkable factor to consider when selecting the stimulation protocol for the first cycle of infertile patients [*P* for interaction: 0.013].

Results of the analysis stratified by female age showed that, in women under 40 years, the cLBRs were comparable between the two groups [female age <30 years: OR (95% CI), 1.01 (0.82, 1.23); female age ≥30 years and <40 years: 0.92 (0.77, 1.10)] (Table 2). Notably, the cLBR remarkably decreased in the GnRH antagonist group in women above 40 years old [OR (95% CI), 0.58 (0.21, 1.58)], although the change was not statistically significant. Results of interaction test indicated that there was no interaction effect between female age and stimulation protocol [*P* for interaction: 0.526].

The study population was further divided into 9 groups according to the combination of AFC and female age (Table 3). The results of multivariable regression analyses showed that in younger patients (<30 years) with low ovarian reserve (AFC ≤ 7), the flexible GnRH antagonist protocol led to significantly lower cLBR than the GnRH agonist long protocol [OR (95% CI), 0.29 (0.11, 0.74)]. However, in younger patients (<30 y) with high ovarian reserve (ACF > 24), the cLBR was significantly higher in the GnRH antagonist protocol group than in the GnRH agonist long protocol group [OR (95% CI), 1.78 (1.07, 2.96)] (Table 3). These results were in consistency with the results shown in Table 2, but the effect value was more robust when the study population was more specific [cLBR in younger patients (<30 years) with low ovarian reserve (AFC ≤ 7): OR (95% CI), 0.29 (0.11, 0.74); cLBR in overall patients with low ovarian reserve (AFC ≤ 7): OR (95% CI), 0.62 (0.41, 0.94); cLBR in younger patients (<30 years) with high ovarian reserve (ACF > 24): OR (95% CI), 1.78 (1.07, 2.96); cLBR in overall patients with high ovarian reserve (ACF > 24): OR (95% CI), 1.43 (0.96, 2.12)]. Moreover, the robustness of the results was further proved by multivariable regression analysis in cohort with missing data, cohort with complete data and cohort with multiple imputation for the missing data (Supplementary Table 1). These results also indicated that the cLBR significantly decreased in the GnRH antagonist group in women under 30 years with low ovarian reserve, and also remarkably decreased in patients above 40 years regardless of AFC. There was no patient over 40 years and with AFC more than 24 at the same time.

**Table 3:** Comparison of cLBR of flexible GnRH antagonist protocol vs. GnRH agonist long protocol using multivariable regression analysis in patients stratified by AFC and age.

	**Non-adjusted**	***P***	**Adjusted**	***P***
**Basal AFC** **≤7**				
Female age <30 y	*0.27 (0.12, 0.59)*	*0.0012*	*0.29 (0.11, 0.74)*	*0.0096*
Female age ≥30 y, <40 y	0.80 (0.50, 1.27)	0.3379	0.87 (0.52, 1.45)	0.5945
Female age ≥40 y	0.74 (0.14, 3.91)	0.7252	0.54 (0.08, 3.38)	0.5069
**Basal AFC** **>7**, **≤24**				
Female age <30 y	1.04 (0.83, 1.30)	0.74	1.02 (0.81, 1.28)	0.8557
Female age ≥30 y, <40 y	1.06 (0.58, 1.97)	0.5685	1.05 (0.85, 1.29)	0.6327
Female age ≥40 y	0.88 (0.26, 2.97)	0.8341	0.51 (0.10, 2.60)	0.4194
**Basal AFC** **>24**				
Female age <30 y	1.52 (0.96, 2.43)	0.075	*1.78 (1.07, 2.96)*	*0.0269*
Female age ≥30 y, <40 y	1.06 (0.58, 1.97)	0.8555	1.01 (0.50, 2.02)	0.9789
Female age ≥40 y	NA		NA	NA

*The italic values represent that the differences are statistically significant*.

## Discussion

The present study compared the cLBRs between flexible GnRH antagonist protocol and the standard GnRH agonist long protocol in which the short-acting GnRH agonist for pituitary down regulation started from luteal phase of the menstrual cycle. Multivariable regression analyses were used for subgroup analysis in patients of different ages and various ovarian reserve. We found that compared with long protocol, flexible GnRH antagonist protocol might not be suitable for patients with basal AFC ≤ 7, especially those younger than 30 years and with low basal AFC, nor for patients over 40 years regardless of AFC, because it resulted in significantly lower cLBR in these subgroups (Tables 2, 3). However, flexible GnRH antagonist protocol appeared beneficial for younger patients (<30 years) with high ovarian reserve (AFC > 24), as it remarkably improved the cLBR in this group of patients (Table 3). While for younger patients (<30 years) with normal ovarian reserve (AFC > 7 and ≤ 24) and patients between 30 and 40 years with basal AFC more than 7, the cLBRs between the two protocol groups were comparable (Table 3).

A number of RCTs have compared clinical outcomes between GnRH antagonist and GnRH agonist protocols in the last few decades. Al-Inany, et al. conducted a meta-analysis comparing the effectiveness and safety of the two protocols based on these RCTs published since 2011 and kept updating the meta-analysis until 2016 (12–14). Seventy-three RCTs and 12,212 participants were included in their latest Cochrane meta-analysis (12). However, no difference was found in LBR comparison between GnRH antagonist and GnRH agonist long protocols in the general population [RR (95% CI), 1.02 (0.85, 1.23); 12 RCTs, *n* = 2,303, *I*^2^= 27%, moderate quality evidence] and quality of the evidence was evaluated as moderate due to poor reporting of study methods. The subgroup analysis was performed for patients receiving the minimal stimulation protocol and different trigger methods but not in populations with different demographic characteristics. In crude comparison of demographic characteristics of the study population in Table 1, lower AMH and AFC were noticed in GnRH antagonist group, this could partially explain the less retrieved oocytes, mature oocytes, less embryos and the lower cLBR in this group. However, after adjusting some risky confounders, such as female age, infertility factors, infertility duration, infertility diagnosis, female BMI and fertilization method, the results of multivariable regression analysis showed similar cLBRs between the two protocol groups [OR (95% CI), 1.00 (0.87, 1.14)] (Table 2) in the general study population, even in the presence of the differences of AMH, AFC and the number of retrieved oocytes. However, the situation significantly changed when the analysis was performed in subgroup patients with different ovarian reserve defined by age and AFC.

In our study, among the population with low ovarian reserve defined as AFC ≤ 7, cLBR was significant lower in the GnRH antagonist group than in the GnRH agonist long protocol group [OR (95% CI), 0.62 (0.41, 0.94)]. The meta-analysis performed by Lambalk et al. (17) comparing the clinical outcomes between these two protocols in poor responders included three RCTs (21–23) with 544 participants, and the pooled estimate of LBR was calculated. The results revealed slightly lower LBR in the GnRH antagonist group but the difference was not statistically significant [RR (95% CI), 0.89 (0.56, 1.41)]. The trend found in our study was in line with the previous studies. The sample size of the three primary articles included in the meta-analysis was small (*n* = 120 (22), 60 (21), and 364 (23), respectively) and the definition of poor responders varied in each study. Of the three RCTs, the RCT by Prapas et al. showed lower but not significantly different pregnancy rate in the GnRH antagonist protocol group (23); Kim et al. found no difference in the LBRs between the two groups (22); and Marci et al. found higher but not significantly different pregnancy rate in the GnRH antagonist protocol group (21), which was not consistent with results of other studies, probably due to the small sample size (*n* = 60). Sample size of the adjusted regression analysis in the low ovarian reserve population in our study was 594, much larger than in the previous RCTs. Xiao et al. (24) also reported similar clinical pregnancy rates [RR (95% CI), 0.79 (0.54, 1.14)] between the two protocol groups in their meta-analysis with a slightly bigger sample size (including 5 articles and 597 poor responders), and the trend of their results appears consistent with ours.

To explore the population-specific application of the COS protocols, we further divided the low ovarian reserve population into three groups according to female age: female age <30 y; >30 y, <40 y; and ≥ 40 y. Our results showed that the GnRH antagonist protocol resulted in significantly and consistently lower cLBR with very robust effect value in younger patients with low ovarian reserve (age < 30 years plus AFC ≤ 7) [OR (95% CI), 0.29 (0.11, 0.74), *n* = 112], indicating that the flexible GnRH antagonist protocol might not be suitable for this population. Furthermore, when patients younger than 30 years and with low ovarian reserve was excluded from the cohort, the result became consistent with the previously reported studies (17, 21–23), that GnRH antagonist resulted in lower cLBR despite the lack of statistical significance. This suggests that younger patients with low ovarian reserve are the main contributors to the decrease of cLBR in low ovarian reserve population using GnRH antagonist protocol. The population of young patients with low ovarian reserve is very similar, categorized as Group 3 according to the POSEIDON criteria, namely patients under 35 years but with diminished ovarian reserve (25). The rationale of the classification criteria is that younger patients with low ovarian reserve might have some specific genetic background or underlying disease so that they appear to be resistant to exogenous gonadotropins and thus may require different treatment (26). A retrospective study in POSEIDON group three patients conducted by Huang et al. found that GnRH antagonist protocol resulted in significantly lower LBR than the GnRH agonist long protocol (GnRH antagonist protocol vs. GnRH agonist long protocol: 16.7 vs. 30.8%) (27), which results were consistent with ours. Moreover, the results of interaction test in our study showed that the ORs for cLBRs among each AFC group were significantly different (*P* for interaction = 0.013), suggesting that AFC might be a potential predictor for outcome of the two protocols. A prediction model built by Broekmans et al. (28) revealed that AFC was one of the common prognostic factors for high and low ovarian response in patients using GnRH antagonist protocol. This result was supported by a retrospective case-control study by Reichman et al. (29) who concluded that after matched for age, patients with premature LH surge had significantly decreased AFC, indicating that the diminished ovarian reserve seems a predominant risk factor for GnRH antagonist protocol failure in terms of premature LH surge in IVF cycles, and the low ovarian reserve might also exaggerate the negative effect of advanced follicular maturation and the asynchronized follicular development involved in GnRH antagonist protocol in this population. With these results, we wouldn't recommend GnRH antagonist protocol as the initial protocol in the first IVF cycle for patients with low ovarian reserve, especially young ones.

Multiple meta-analyses have compared clinical outcomes between the GnRH agonist long protocol and the GnRH antagonist protocol in PCOS patients with high ovarian reserve (AFC > 24) and found no difference in clinical pregnancy rate between the two protocols (15–17). Interestingly, our study showed that the cLBR varied significantly between different age groups. For young patients with high ovarian reserve (age < 30 years, AFC > 24), GnRH antagonist protocol resulted in significantly higher cLBR [OR (95% CI), 1.78 (1.07, 2.96), *n* = 336], while for patients between 30 and 40 years and with high ovarian reserve (AFC > 24), GnRH antagonist protocol resulted in similar cLBR as the GnRH agonist long protocol did [OR (95% CI), 1.01 (0.50, 2.02), *n* = 198]. No patient was above 40 y and also had more than 24 AFC in the current cohort. These results suggest the positive effect of flexible GnRH antagonist protocol in young patients with high ovarian reserve due to the increased cLBR and decreased OHSS rate according to previous reports (9, 15, 17). In addition, in patients aged between 30 and 40 years and with high ovarian reserve (AFC > 24), GnRH antagonist protocol is still favored due to the comparable cLBR but significantly decreased OHSS rate when compared with the GnRH agonist long protocol according to previous reports (9, 15, 17)

Notably, for patients above 40 years, cLBR in the GnRH antagonist group was lower although without statistical significance, the effect value showed robustness [OR (95% CI), 0.58 (0.21, 1.58)], and this trend was not affected by AFC [cLBR in patients above 40 y with AFC ≤ 7: OR (95% CI), 0.54 (0.08, 3.38); cLBR in patients above 40 y with AFC > 7 and ≤ 24: OR (95% CI), 0.51 (0.10, 2.60)]. Unfortunately, to the best of our knowledge, there are no studies specifically comparing effectiveness of the GnRH agonist long protocol and the GnRH antagonist protocol in women of advanced age. Result of our study suggest that for infertile women above 40 years, regardless of their AFC, flexible GnRH antagonist protocol should not be recommended as the COS protocol in the first cycle, because it would remarkably decrease the cLBR in this population.

For patients with normal ovarian reserve (AFC >7, ≤ 24) and under 40 years, GnRH antagonist protocol would be favored since it would result in comparable cLBR [cLBR in patients under 30 y with AFC >7, ≤ 24: OR (95% CI), 1.02 (0.81, 1.28); cLBR in patients between 30 and 40 years with AFC >7, ≤ 24: OR (95% CI), 1.05 (0.85, 1.29)] and significantly decreased OHSS rate compared with the GnRH agonist long protocol according to previous reports (9, 15, 17). The recommendations for clinical application of the standard GnRH agonist long protocol and the flexible GnRH antagonist protocol tailored by age and AFC is schemed in Supplementary Table 1.

There are two major limitations in the present study. Firstly, this is a retrospective study with some intrinsic limitation such as potential selection bias despite all the efforts to control it (30). The second limitation is that OHSS occurrence data was not collected, while OHSS rate is an important indicator for safety of ART (31). However, we collected data on the number of embryo transfer cycle cancelation for OHSS prevention. Although the data was incomplete with missing data in 1,263 cycles, we could preliminarily tell that the number of embryo transfer cycle cancelation due to OHSS risk was significantly less in GnRH antagonist group than in the GnRH agonist long protocol group (Table 1). The result is consistent with the previous reports (9, 15, 32), and to some extent, provides evidence in favor of GnRH antagonist protocol in younger patients (<30 years) with normal ovarian reserve (AFC >7, ≤ 24) and in patients between 30 and 40 years with basal AFC more than 7 in the present study, in which, the cLBR were comparable between the two protocol groups.

## Conclusion

The results of the study indicate that the GnRH antagonist protocol might not be suitable for patients with low ovarian reserve (AFC ≤ 7) or patients over 40 years old because it would result in significantly lower cLBR in these patients, and the negative effect seems even more robust in patients under 30 years and with low ovarian reserve (AFC ≤ 7). However, flexible GnRH antagonist protocol might be strongly recommended for patients under 30 years and with high ovarian reserve (AFC > 24) due to the significantly increased cLBR in this group of patients. For the rest of patient groups in the present cohort, the cLBR was comparable between the two protocols; while antagonist protocol would still slightly be favored because it resulted in lower OHSS rate in general population and in PCOS population according to previous reports (9, 15, 17). The recommendation intensity of the two COS strategies in different populations is shown in Supplementary Figure 1.

## Data Availability Statement

All datasets generated for this study are included in the article/Supplementary Material.

## Ethics Statement

The studies involving human participants were reviewed and approved by Institutional Review Board, The Second Affiliated Hospital of Air Force Medical University. Written informed consent for participation was not required for this study in accordance with the national legislation and the institutional requirements.

## Author Contributions

XW, WZ, and DX contributed conception and design of the study. WZ conducted the statistical analysis and wrote the first draft of the manuscript. DX organized the database. HZ, JH, and BW performed the clinical procedure. XX wrote sections of the manuscript. YT and YM did the follow-up of the patients. All authors read and approved the final paper.

## Conflict of Interest

The authors declare that the research was conducted in the absence of any commercial or financial relationships that could be construed as a potential conflict of interest.
