# Supplementary figures and images for: Cumulative Live Birth Rates After the First ART Cycle Using Flexible GnRH Antagonist Protocol vs. Standard Long GnRH Agonist Protocol: A Retrospective Cohort Study in Women of Different Ages and Various Ovarian Reserve
Source: Front Endocrinol (Lausanne). 2020 May 8;11:287. doi: 10.3389/fendo.2020.00287 (PMC7225261; doi:10.3389/fendo.2020.00287)

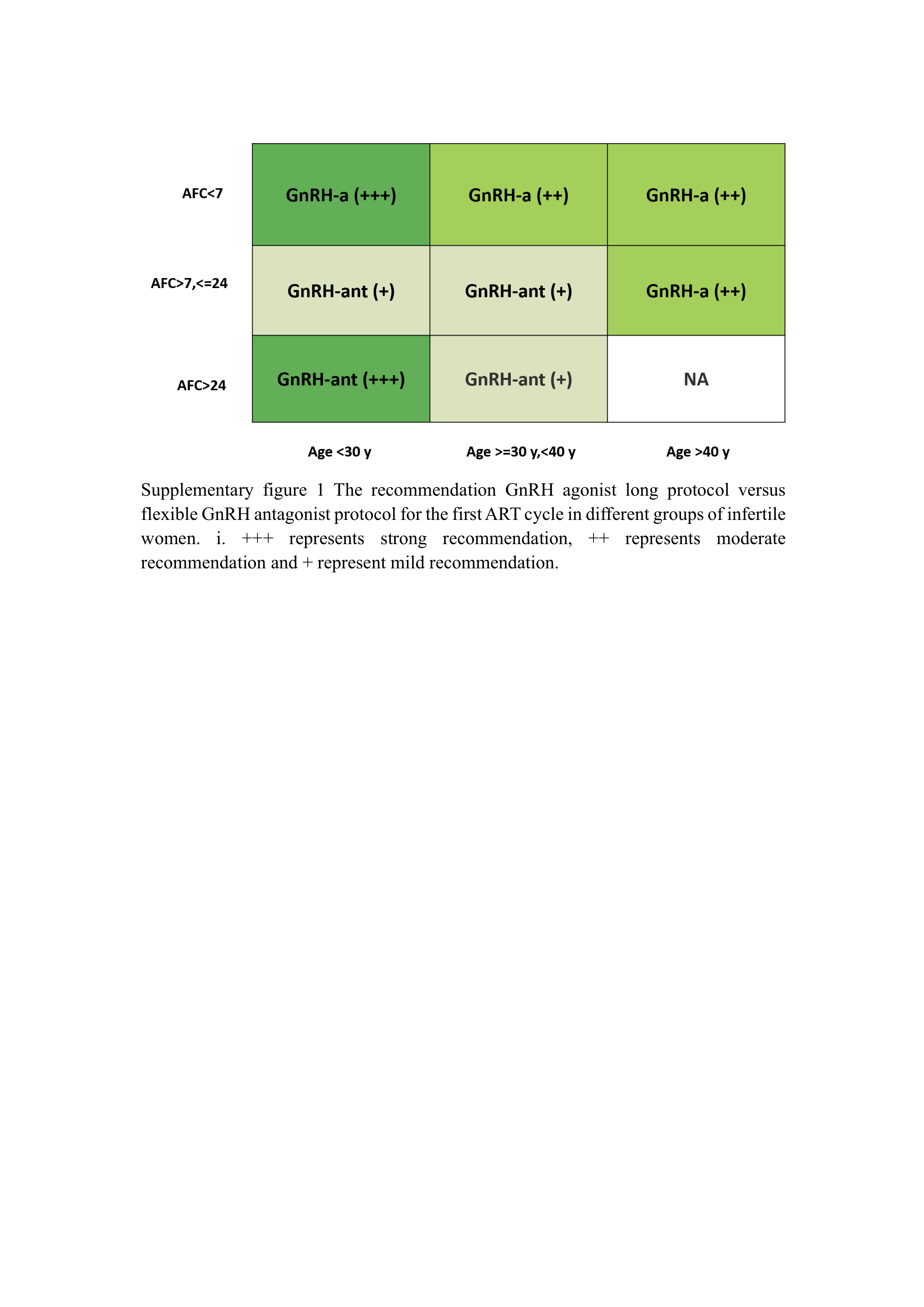

Supplement: Supplementary file 2 [file Image_1.TIFF]
